# Swyer-James-MacLeod Syndrome: A Rare Cause of Unilateral Hyperlucent Lung in a Two-Year-Old Male Child

**DOI:** 10.7759/cureus.103485

**Published:** 2026-02-12

**Authors:** Sarika Gupta, Himanshu Gupta, Sakshi Singh

**Affiliations:** 1 Department of Paediatrics, King George's Medical University, Lucknow, IND

**Keywords:** bronchiolitis obliterans, hypovascular lung, pediatric pulmonary disorders, swyer james macleod syndrome, unilateral hyperlucent lung

## Abstract

Swyer-James-MacLeod syndrome (SJMS) is an uncommon post-infectious bronchiolitis obliterans-related pulmonary disorder characterized by a unilateral hyperlucent lung with associated hypovascularity on chest radiography and CT, and markedly reduced perfusion on ventilation-perfusion scintigraphy. Although typically identified in older children or adolescents due to its insidious progression, diagnosis in early childhood remains rare and diagnostically challenging.

This report describes a two-year-old male child with recurrent lower respiratory tract infections who was ultimately diagnosed with SJMS based on characteristic radiologic hallmarks. This case emphasizes the importance of maintaining a high index of suspicion for SJMS in young children presenting with persistent or unexplained unilateral pulmonary abnormalities. It highlights the critical role of multimodal imaging in early recognition.

## Introduction

Swyer-James-MacLeod syndrome (SJMS) is a rare chronic pulmonary disorder that represents a long-term sequela of post-infectious bronchiolitis obliterans [1], most commonly following severe viral lower respiratory tract infection in early childhood, particularly adenovirus infection. The condition is characterized by a unilateral hyperlucent lung on imaging, resulting from persistent air trapping, impaired alveolar development, and hypoplasia of the pulmonary vasculature [1-3].

Although the inciting infection usually occurs during infancy, the diagnosis of SJMS is frequently delayed until late childhood or adulthood. This delay is attributed to the nonspecific nature of clinical symptoms and the gradual evolution of radiological features as lung growth occurs. Patients may present with recurrent respiratory infections, chronic cough, wheezing, or exertional dyspnea, while some remain asymptomatic and are diagnosed incidentally.

Early diagnosis of SJMS is uncommon, particularly in children younger than five years of age, as recurrent wheezing is often misattributed to asthma or viral bronchiolitis. Failure to recognize this condition early may lead to inappropriate long-term therapy and missed opportunities for targeted follow-up and prevention of complications such as bronchiectasis and pulmonary hypertension [2,4]. Cross-sectional imaging, especially computed tomography, plays a crucial role in early identification by demonstrating characteristic features such as air trapping and reduced pulmonary vascularity.

The objective of this case report is to highlight the possibility of Swyer-James-MacLeod syndrome presenting in early toddlerhood, emphasize the importance of considering this rare entity in children with recurrent respiratory symptoms and unilateral radiological abnormalities, and underscore the role of early imaging in establishing a diagnosis.

By reporting this case, we aim to increase awareness among pediatricians and radiologists, facilitate timely diagnosis, and promote appropriate long-term management strategies.

## Case presentation

**Figure 1:**
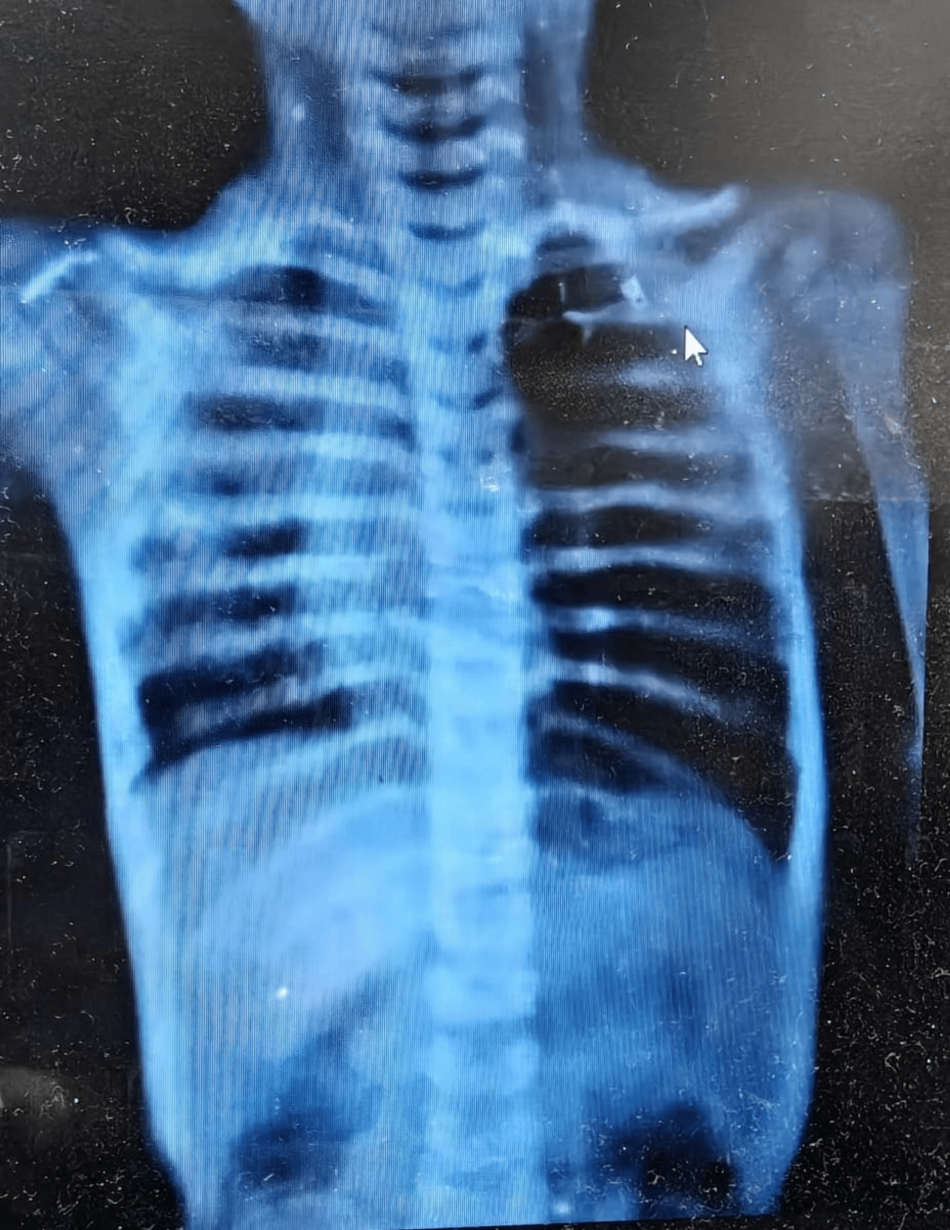
Chest X-ray anteroposterior view Marked unilateral hyperlucency of the left lung with reduced vascular marking in the left lung.

A two-year-old male child presented with a history of recurrent cough, episodic wheezing, and exertional breathlessness persisting since approximately eight months of age. On examination, the child was tachypneic with mild subcostal retractions and maintained an oxygen saturation of 94% on room air. Auscultation revealed reduced breath sounds over the left hemithorax with intermittent expiratory wheeze, while the remainder of the systemic examination was within normal limits. There was no family history of asthma, tuberculosis, or other chronic respiratory illnesses. A chest X-ray revealed marked unilateral hyperlucency of the left lung with reduced vascular markings (Figure 1). 

**Figure 2:**
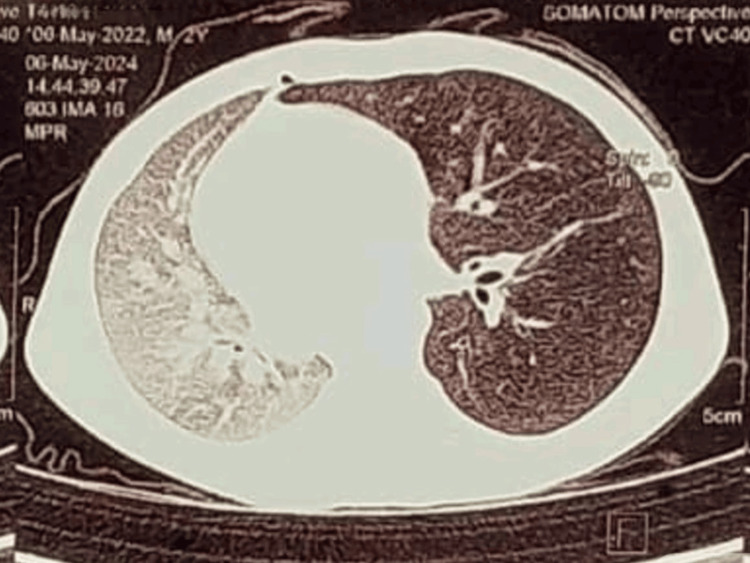
Contrast-enhanced CT thorax, axial view Window for pulmonary parenchyma in axial view showing overinflation of the left lung and midline herniation, accompanied by a contralateral tracheal and mediastinal shift.

**Figure 3:**
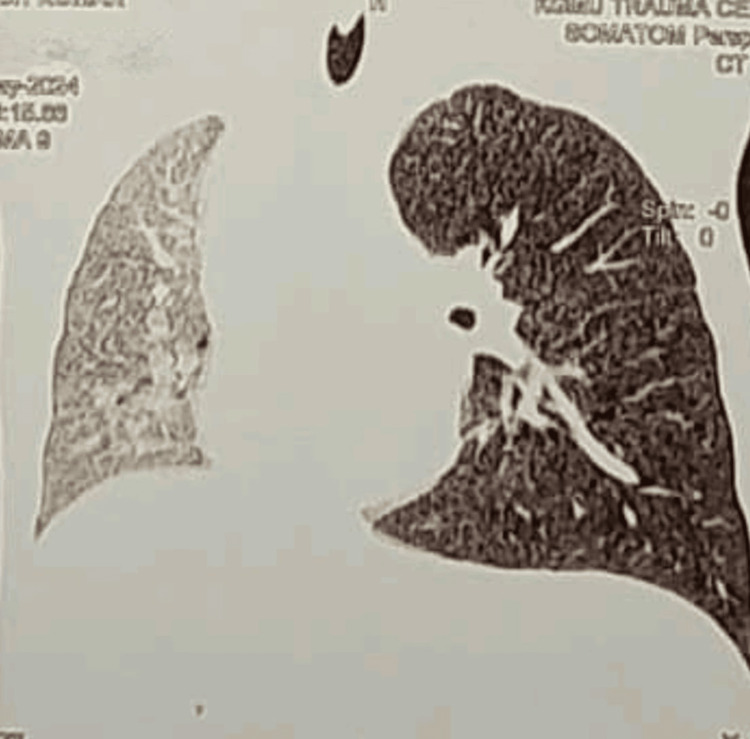
Contrast-enhanced CT thorax, coronal view Window for pulmonary parenchyma in coronal view showing ground glass attenuation diffusely in the right lung with overinflation of the left lung.

**Figure 4:**
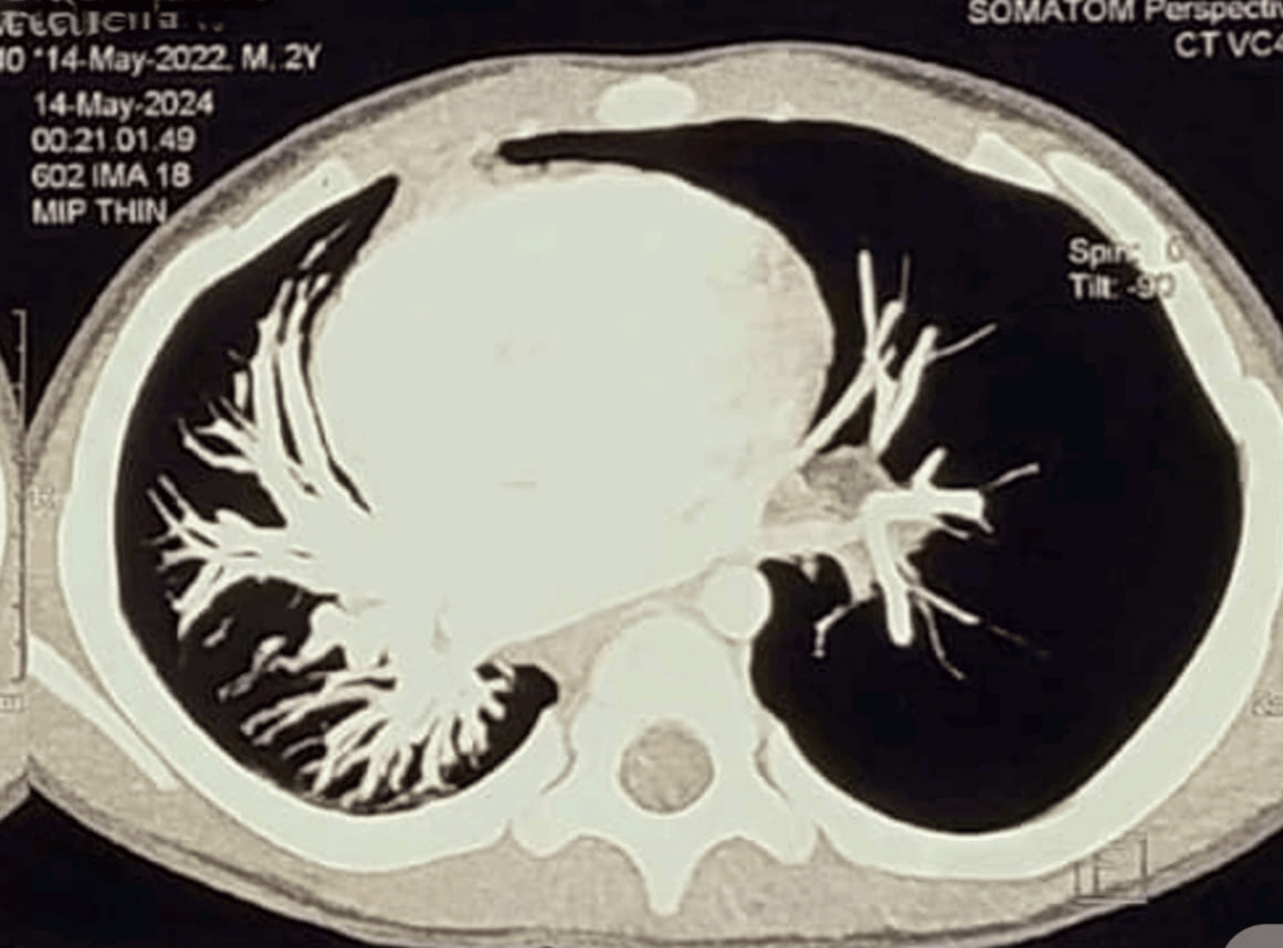
Pulmonary angiography maximum intensity projection (MIP) image The left segmental pulmonary artery is smaller in caliber with attenuating branches.

**Figure 5:**
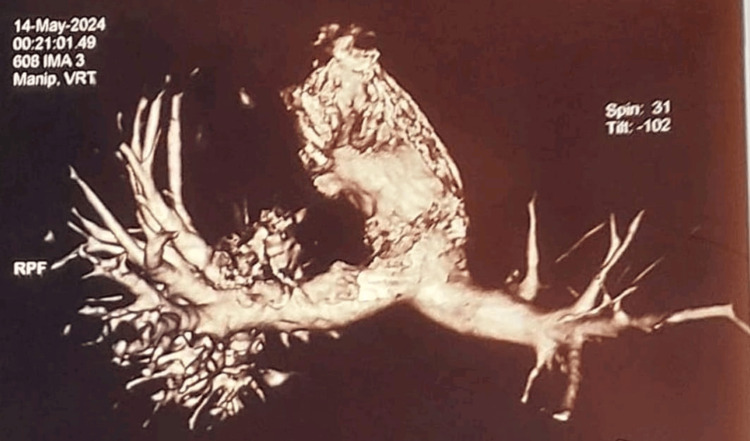
Pulmonary angiography maximum intensity projection (MIP) image with 3D reconstruction The left segmental pulmonary artery is smaller in caliber with attenuating branches.

**Figure 6:**
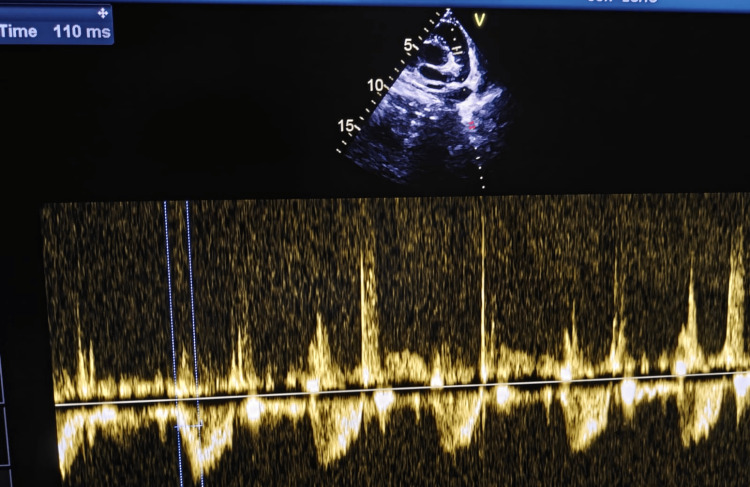
2D echo: high parasternal short axis (PSAX) view Mean pulmonary artery pressure (mPAP) = 90 − (0.62 × acceleration time) mPAP = 90 − (0.62 × 110) = 21.8 mmHg, i.e., normal (Normal mPAP in children >3 months is <20–25 mmHg).

Contrast-enhanced CT (CECT) of the thorax demonstrated overinflation of the left lung and midline herniation, accompanied by a contralateral tracheal and mediastinal shift, findings characteristic of Swyer-James-MacLeod syndrome (Figures 2, 3). Pulmonary angiography showed a hypoplastic left segmental pulmonary artery with attenuated peripheral branches, supporting the diagnosis of unilateral hypovascular lung consistent with SJMS (Figures 4, 5). Echocardiography revealed normal findings, with no evidence of pulmonary hypertension (Figure 6). 

Based on the clinical presentation and characteristic radiologic and angiographic findings, a diagnosis of Swyer-James-MacLeod syndrome was established.

The child was managed conservatively with inhaled bronchodilators and corticosteroids to optimize airway patency and reduce inflammatory exacerbations. Chest physiotherapy was initiated to enhance airway clearance and prevent mucus plugging. Age-appropriate immunizations, including influenza and pneumococcal vaccines, were administered to minimize the risk of future respiratory infections.

The parents were counseled on environmental control measures, such as avoiding indoor air pollutants, allergens, and passive smoke exposure, and on the importance of prompt medical evaluation during respiratory illnesses to prevent further airway injury.

During a six-month follow-up period, the child demonstrated clinical improvement with a significant reduction in the frequency and severity of respiratory infections. Surgical intervention was not considered, as the disease involvement was diffuse and pulmonary function remained adequately preserved.

## Discussion

SJMS is a rare chronic pulmonary condition that represents a late sequela of post-infectious bronchiolitis obliterans. It results from inflammatory injury to the terminal and respiratory bronchioles following severe lower respiratory tract infection in early childhood, most commonly caused by adenovirus. The ensuing fibrosis and obliteration of small airways lead to persistent air trapping, impaired lung growth, and secondary hypoplasia of the pulmonary vasculature on the affected side [5].

Adenovirus, particularly serotypes 3, 7, and 21, is the most frequently implicated pathogen, although Mycoplasma pneumoniae and other viral agents have also been associated with disease onset [6,7]. SJMS is typically diagnosed in late childhood or adolescence, and its occurrence in a child as young as two years is therefore unusual.

The hallmark radiological feature of SJMS is a unilateral hyperlucent lung with reduced vascular markings. Computed tomography plays a pivotal role in diagnosis by demonstrating areas of air trapping, best appreciated on expiratory images, along with diminished caliber and number of pulmonary vessels [2,5]. In advanced cases, associated findings such as bronchiectasis, mosaic attenuation, and volume loss may be present. In our patient, the absence of bronchiectasis likely reflects early-stage disease, emphasizing the benefit of early diagnosis.

SJMS is typically diagnosed in late childhood or adulthood, often incidentally during imaging performed for unrelated reasons. Presentation in early toddler age, as seen in this case, is distinctly uncommon. Early diagnosis can be challenging because clinical manifestations, such as recurrent wheezing and respiratory infections, often mimic more common pediatric conditions, including asthma or recurrent viral bronchiolitis. This frequently leads to delayed recognition and inappropriate long-term treatment. A high index of suspicion is therefore required when recurrent respiratory symptoms are associated with unilateral radiological abnormalities.

The differential diagnosis of unilateral hyperlucent lung in children is broad and includes congenital lobar emphysema, pulmonary hypoplasia, bronchial foreign body, post-tubercular sequelae, and pulmonary artery anomalies. Congenital causes typically present earlier in life and often lack a history of severe lower respiratory tract infections. Foreign body aspiration typically presents with an acute onset and localized hyperinflation, whereas post-tubercular disease is usually associated with parenchymal scarring and lymphadenopathy. Careful clinical correlation and characteristic CT findings help differentiate these entities from SJMS.

Management of SJMS is primarily conservative and supportive. The cornerstone of treatment includes prevention of respiratory infections through immunization, prompt antibiotic therapy during infectious exacerbations, bronchodilators for symptomatic relief, and chest physiotherapy to enhance airway clearance. Surgical intervention, such as lobectomy or pneumonectomy, is rarely indicated and is reserved for patients with severe localized disease complicated by recurrent infections, persistent hyperinflation, hemoptysis [3,4], or severe bronchiectasis. Long-term follow-up is essential to monitor for potential complications, including progressive bronchiectasis, recurrent pneumonia, and pulmonary hypertension [8].

The prognosis is generally favorable when recurrent infections are controlled and adequate lung function is maintained.

This case underscores the importance of recognizing SJMS at an early age, particularly in children with a history of severe pneumonia in infancy and persistent unilateral radiological abnormalities.

Early diagnosis enables appropriate counseling for caregivers, avoidance of unnecessary investigations or prolonged asthma therapy, and the institution of preventive strategies to preserve lung function.

## Conclusions

SJMS is a rare and often under-recognized cause of unilateral hyperlucent lung in early childhood, particularly in children younger than the typical age of presentation. This case highlights the importance of considering SJMS in young patients with recurrent respiratory symptoms and persistent unilateral radiologic abnormalities. Early diagnosis through appropriate multimodal imaging, especially HRCT and angiography, facilitates timely initiation of supportive therapy and preventive measures. Conservative management, including bronchodilators, inhaled corticosteroids, chest physiotherapy, vaccination, and environmental control, can lead to significant clinical improvement and reduce the risk of long-term pulmonary impairment. Awareness of this entity among pediatricians and radiologists is essential to prevent misdiagnosis, unnecessary interventions, and disease progression.
